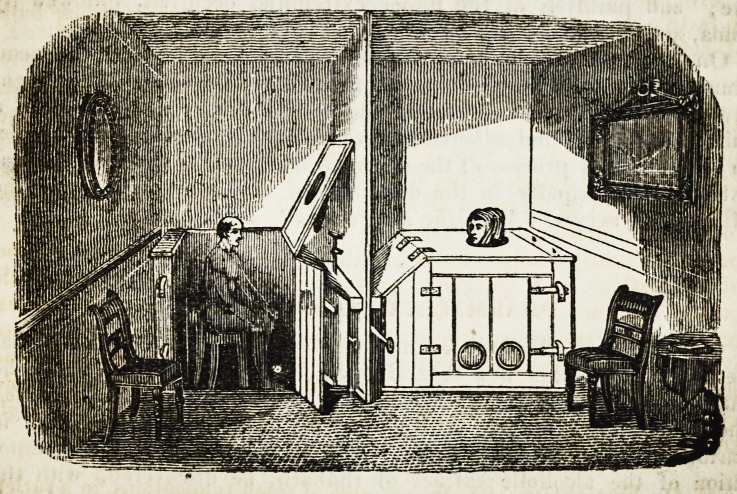# Medical and Physical Intelligence

**Published:** 1826-03

**Authors:** 


### MEDICAL AND PHYSICAL INTELLIGENCE.

### PATHOLOGY.

1. *Hydrophobia.*—In a Memoir lately presented to the Academy, by M. Naudin, of Toulouse, a case of Hydrophobia is related. It appears that six persons were bitten by a dog not supposed to be rabid, and consequently no precautions were taken: forty-eight days afterwards, symptoms of hydrophobia appeared in one of the persons bitten, and death ensued in two days. The pustules described by Marochetti *were not discovered,* but the sublingual glands were in a state of inflammation, and they were cauterised without success. The other five persons had experienced no inconvenience at the time this account was given, one month after the death of the above patient. (*Archives Generales*, Decembre.)

2. *Case of Enlargement of the Mammæ.*—M. Delfix, of Morlais, has published a paper in the Journal of Physiology, on the use of Hydriodate of Potash, from which we extract the following case:—

Maria Ahaure, residing in the Lower Pyrennees, thirty years of age, of a lymphatic temperament, consulted him, in the year 1821, for a venereal affection. The use of mercurial frictions soon restored her to health. Two years afterwards, she again consulted the Doctor for a similar disease, and also on account of her breasts, which were enlarged: they were three times their natural size. The liquor of Van Swieten (solution of corrosive sublimate,) was prescribed, which, with other means, produced a diminution of all the syphilitic symptoms, but the mammæ still continued to increase. This augmentation was considered to be a venereal symptom, and mercurial frictions were employed under the arms, until an ounce of the ointment was used; but, instead of procuring any amendment, the breasts continued to increase to a most extraordinary degree. When the patient was sitting, they rested upon her thighs; they could be joined together behind her back; the nipples were like hen's eggs. The patient, however, experienced no pain, and the skin retained both its suppleness and colour. Unknown to Dr. Delfiz, a surgeon had plunged his bistoury deeply into this mass, in the expectation of evacuating pus; but only a few drops of blood followed the incision. The patient declared that she scarcely felt this operation. At this period she had miscarried, in the fifth month of her pregnancy.

Recollecting the good effects of the hydriodate of potass in bronchocele, as well as that it had been found useful in some presumed syphilitic swellings, the Doctor determined upon making trial of it. In consequence, frictions with half an ounce of the pommade, made according to the formulary of M. Magendie, were recommended; emollient fomentations being employed at the same time. No accident occurred; and this treatment was continued for a month and a half. At the end of this time, the breasts were much diminished in size. After an intermission of fifteen days, the pommade was again had recourse to, and employed for three months, the frictions being repeated less frequently; otherwise, the patient complained of headache. The result was, that the breasts were reduced to about twice their natural size: they were flaccid; and the woman was able to undergo severe labour. (*Journal de Physiologie*, Octobre.)

3. *Necrosis.*—Dr. RICHTER, of Berlin, has lately published some long and interesting papers upon the subject of the various kinds of Necrosis. We abstract the following observations, which may not be uninteresting to our readers.

*Necrosis syphilitica.*—This species begins mostly in the substance of the bones, and extends lengthways. The pain (which may last for a considerable time) is of a boring, gnawing kind, and increases when the patient is warm in bed. At this time, indeed, it frequently is so severe, that he is obliged to rise. The swelling gradually increases, but does not acquire the size which is witnessed when the disease arises from other causes. The inflammatory process is slowly developed, never very acute, and does not extend beyond the part of the limb which has been previously swoln. The redness is of a pale rose-colour, very limited, well defined, and not gradually lost in the surrounding parts. The temperature of the part is not high. The pain arising from inflammation of the soft part is more superficial and lancinating, and may be distinguished, by intelligent patients, from the boring, gradual, and deeper-seated pain, which is characteristic of this form of necrosis. The swelling of the limb is but little increased when inflammation of the skin supervenes, and every symptom is increased when the patient is warm in bed. Some time elapses before an opening takes place in the skin; and, before the small portion of skin is absorbed, it assumes a limited dark appearance. Subsequently, the skin in the neighbourhood of this opening is more destroyed than the cellular membrane beneath. When necrosis arises from other causes, the latter structure is most affected. Around the opening small superficial ulcerations form, which take on all the characters of chancres. The matter, which is from the commencement discharged from these openings in the skin, differs much from the qualities of healthy pus, particularly if the patient has been long affected with syphilis, and is of a cachectic habit of body.

*Necrosis arthritica* occurs only in advanced age. It arises either suddenly or gradually, and may show itself, at first, either upon the surface, or the periosteum, or the joints, of the bones themselves. Its course is rapid, if the subject is of a full plethoric habit of body. The paroxysm of gout is, probably, suddenly suppressed. In patients of a different constitution, it proceeds more slowly. The pain is acute, and seldom felt in the middle of the bones, but more superficially upon the periosteum, and affects the whole circumference of the limb equally. In this species, warmth is borne without any aggravation of the symptoms; but, towards midnight, there is generally an exacerbation, the severity of which passes off in the morning. Upon any approaching change of weather, the pain is much increased. The swelling quickly follows the pain, and soon becomes considerable, as the inflammation begins mostly in the soft parts, and subsequently extends to the bones.

The inflammation sometimes takes place at the same time with the swelling, and extends to the same distance. The redness is darker than that observed in the preceding species, and less diffused; but still gradually vanishes towards the edges. The sensibility of the inflamed part is often so considerable, that the slightest covering cannot be borne. The fistulous openings are small, and the edges easily become callous. The fluid at first discharged is thin and watery, and excoriates the adjacent parts: it sometimes, under circumstances of a favourable nature, becomes of a better quality.

*Necrosis scrofulosa* takes place, in most cases, during the period of growth, in those subjects who are of a generally scrofulous habit. It arises and proceeds very gradually, and may last for years, affecting at the same time several bones. The pains, at the commencement, are moderate, and observe no regular course: they are not increased when the patient is warm in bed, nor by vicissitudes of weather. The swelling also takes place very slowly, but extends over the whole of the affected parts; perhaps occupies all the limb, the under part being œdematous. It becomes gradually larger, and is not tense and hard, but remains of a doughy feel. In this state it may remain, the pain continuing for some time before inflammation supervenes. At last inflammation does take place, but does not rise to a high degree; the part appearing of a pale rose-colour, as in the syphilitic species: it is more diffused, however. When the disease is of long duration, and a great part of the bone is destroyed, the colour of the inflamed part becomes of a bluish cast, and darker. It is still distinguished by its asthenic character, by the swelling of the part, which remains doughy, and retains the impression of a finger. The fistulous openings possess the peculiarity of easily healing, and again breaking out; or the passage to the destroyed portion of bone is closed up by fungous growths, which shoot from the openings. The matter at first discharged has the colour and consistence of good pus, but is often mixt with shreds resembling the coagulated white of an egg. The pains frequently abate entirely after the swelling has broken.

*Necrosis scorbutica.*—Besides the general symptoms of weakness and broken health, those which refer to the state of the bone are marked by the following peculiarities:—This species frequently supervenes upon other diseases of the bones; and, long before any destruction of the bone occurs, pain and other symptoms take place. The swelling is violent, and becomes œdematous, and is not for some time accompanied by inflammation; which at last, however, occurs very rapidly. The blush of colour is dark, bluish, and gradually lost in the surrounding parts. The sensibility of the parts, and the temperature, are not so highly increased as in the nervous inflammation. The fistulous openings form rapidly, become large, and approach each other. The sinuses are thin and flaccid, and are disposed to mortify. The matter poured out when the swelling bursts is thin, watery, brown in colour, and mingled with blood: it at last, perhaps, assumes a more favourable appearance. At the edges of the openings, light bleeding fungi form, which increase in size, and obstruct the exit of the contained matter. The contiguous veins are much increased in size.

*Necrosis mercurialis* resembles very nearly the scorbutic species. It runs a tedious and unfavourable course. The general symptoms of the mercurial poison give the clearest indication of the nature of the malady.

*Necrosis metastatica.*—The soft parts surrounding the bones are usually first attacked in this species. The affection of the bones is secondary and sympathetic. The symptoms resulting from a metastatic affection, whether the hard or soft parts are affected, are sudden in occurrence, and rapid in their progress. Pain precedes the other symptoms but a short time: almost along with it arise swelling, inflammation, and abscesses; so that in a few days, or perhaps in a few hours, a portion of destroyed bone may be detected with a probe. The pain occurs suddenly, is very violent, darting, and constant. The swelling is considerable; increases rapidly when the soft parts are primarily affected, and more slowly when the substance of the bones is the seat of the evil. The metastatic inflammation is always limited, violent, and very painful. The redness of a deep tint; the temperature high. The abscess forms, and ruptures speedily. When the muscles and cellular membrane are at first attacked, one opening only is usually formed, and not several at distant points; which occurs more frequently during the development of the disease in the bony structure. The pus discharged is of a bad quality, brown, bloody, of a blackish colour, and thin.

*Necrosis a causa mechanica.*—The cause of this species depends upon the degree of violence inflicted. The progress is generally rapid. The symptoms are violent; and therefore correspond, to a certain degree, with those above described. The nature of the inflammation, and the result of the case, depend almost entirely upon the constitution of the patient. (*Journal für Chirurgie, &c.* von Graefe und Walther.)

We may, in a subsequent Number, abstract a few more observations from the excellent papers of Richter, upon the subject of Necrosis. A continuation of them is promised; and we shall, of course, submit to our readers the treatment proposed by this eminent surgeon in this formidable disease.

### THERAPEUTICS.

4. *Neuralgia of the Sciatic Nerve.*—M. Reveillé Parise has published a paper on the above complaint, in which, after detailing some ineffectual trials with the oil of turpentine, he declares his belief that the method of cure recommended by the celebrated Cotugno, is the only one to be relied upon. To illustrate his meaning, he relates several cases; of one or two of which we here give a sketch. Our own impression, however, being that they were rheumatic affections, and not neuralgia, properly so called.

M. D—, fifty-two years of age, was attacked, without any apparent cause, with sciatica in the limb of the right side, which was for a short time relieved by leeches and rest; but, on resuming his occupation, the pains returned to such a degree, as to render his limb nearly useless. The oil of turpentine was then administered, first in the dose of one drachm a-day, afterwards gradually augmented to three drachms in the day. At first, this medicine seemed to have a beneficial effect; but, when the patient began to exercise the limb, the pain returned with great severity. Our author then applied a large blister to the outer and lower part of the knee, upon the head of the fibula. It was only after a lapse of eight days, that a marked amendment was perceptible; by the twentieth day, the patient was entirely cured, and has remained so. It is to be observed, that costiveness is asserted always to attend this complaint, and that no curative measures will be available unless this state of the bowels be obviated.

M. L—, fifty-seven years of age, was seized with sciatica, in consequence of sitting upon a cold stone seat, in the month of April, 1825. Cupping and repose, with laxatives, effected a cure in about a fortnight. Three months afterwards, in consequence of bathing, M. L— was again attacked with the disease in a very violent degree: the means before resorted to were again tried, but without any good effect. The oil of turpentine, administered to the extent of from three to four drachms a-day, produced but little relief, and brought on a sensation of heat at the epigastrium, and colic. The blister was then applied in the same situation as in the preceding case, and kept moderately open for thirty-two days, when the cure was completed.

From these cases our author draws the following conclusions:—

1st. That the Cotugno method is the most efficacious for the cure of sciatica, especially when it is chronic.

2d. That the suppuration of the blisters or moxas should be kept up for a considerable time.

3d. That, nevertheless, all irritating applications to the ulcers made by the blisters, should be avoided.

4th. That a laxity of the bowels is a necessary condition in the cure of this species of neuralgia; and,

5th. That, in order to avoid relapses, every means must be employed to fortify the diseased limb. Among these means are frictions, baths of heated sand, &c. (*Archives Generales*, Decembre.)

5. ***Further Observations on the Oil of Euphorbia Lathyris.***—M. Bally, at a late sitting of the Royal Academy of Medicine, gave an account of several experiments made by him, at the Hospital la Pitié, upon the action of the above oil. That which he employed had been extracted by means of alcohol, or by expression: this last appeared to be rather more active. Given to fifteen persons of different ages, it produced nearly similar effects; neither did it cause any great number of evacuations. Its purgative power is much inferior to that of the croton tiglium; the dose must be at least doubled, and may be extended from six to ten drops: it has, besides, the defect of exciting vomiting. On the other hand, it does not possess the power of producing salivation, as the croton oil does. M. Bally still, upon the whole, considers it as a useful purgative, and especially for children. It ought to be fresh. (*Ibid.*)

6. ***On Fumigation.***—As the practice of using fumigations, in a variety of complaints, is one of growing utility, we have given a sectional or divided view of two Fumigating Baths, as constructed at Mr. GREEN's establishment for their administration. This view will convey a more distinct idea to many of our country readers, of the nature of a fumigation, than a mere description. The wooden box, represented in the plate, being a very imperfect conductor of heat, is capable of having its temperature increased inside to the degree required for the fumigation,—viz. from 100 to 130° of Fahrenheit; which is occasioned by the construction of the furnace, and hot plates situated beneath the box and under the feet of the patient. On one of these hot plates of iron, the medicine intended for the fumigation is volatilised, and the gas rising surrounds the patient's body, which is previously, (by sitting in this increased temperature eight or ten minutes,) brought to a state favourable to receive the medical agency of the gases produced; as the absorbent system, together with all the functions of the body, are urged into activity by the increased temperature to which the patient is subjected.

Of these gaseous fumigations, we, without hesitation, affirm that they are of the greatest benefit in various disorders of the skin, in cases of indolent ulceration on the surface, and in syphilitic cachexies. On the other hand, in cases of weakened or irregular circulation, in obstruction of the liver, glands, or other viscera, we think equal good is derived by the simple immersion of the patient's body in the heated bath, independently of the gases; for, as this process calls into action the whole of the circulating fluids, it is reasonable to infer that an agent of this nature is a manifest improvement to our therapeutic efforts. At all events, it is an addition that has proved its own recommendation, and does not militate or interfere with our other curative means. In leucophlegmatic habits, likewise, and in the languid circulation of elderly people, this is a remedy that may be beneficially adopted. (*From a Correspondent.*)

### SURGERY.

7. ***Blennorrhagia***, (*Gonorrhœa.*)—MM. Cullerier, the nephew, and Lagneac, made a Report to the Academy upon a Memoir by M. Tarbes, of Toulouse, relative to the means proper to be employed for the purpose of re-establishing the above discharge, when suddenly suppressed. M. Tarbes relates four cases: in the three first, the discharge from the urethra was restored by inoculation of the gonorrhœal matter, in order to overcome in the one case an ophthalmia, and in the two others a swelled testicle. The fourth case relates to an inflammation of the right testicle, produced by the same causes, and which gave way as soon as the discharge was brought back by ammoniacal injections. The reporters appear to think that the inoculation of the matter has no advantage over the mere mechanical irritation of a bougie, or an injection; and they are, moreover, of opinion that, in many instances, the suppression of the discharge, instead of a cause, is really an effect of the new disease. (*Archives Generales*, Decembre.)

8. *Extirpation of the Eye.*—A fungous tumor having developed itself in the right orbit of a girl, seven years of age, who had never shown any previous signs of disease, increased regularly, but without pain, headache, or vomiting, until the eye was pushed out of its natural situation. On the ninth week of the disease, the eye, as well as the fungus, was removed. One month after the operation, a fungous excrescence formed in the orbit, which bled a good deal: after it was removed, it was discovered to be a portion of the cortical substance of the brain. On the succeeding days, violent inflammation of the encephalon, with delirium and obstinate vomiting, came on. A few days afterwards, a fresh excrescence appeared, and increased to such a size as to touch the lips. The same appearances next took place in the left eye; and paralysis of the upper extremities occurred, followed by coma, and death.

On opening the body, an extravasation was found between the membranes of the brain, the substance of which was softened. The fungus in the orbit was flaccid and fetid: the root of this vegetation was of a cancerous nature, and adhered to several bony surfaces; for example, to the pterygoid process of the sphenoid bone and to the sella turcica, extending principally in the direction of the ramifications of the fifth pair of nerves. (*Mag. für die gesammte Heilkunde.*)

---

### PHARMACEUTICAL CHEMISTRY.

9. *Analysis of Rhubarb.*—Some time ago, M. Nani believed that he had discovered a new vegetable alkali in rhubarb, and which he said was capable of being crystallised. M. Caventou has repeated the experiments of the Italian chemist, and regards them as, in various particulars, inaccurate. On examining into the composition of the alcoholic extract of rhubarb, he discovered, with the assistance of alcohol and ether, sometimes separate, at others combined, a fatty matter, containing a little odoriferous volatile oil, and a yellow colouring principle, capable of crystallisation, and of being sublimed without decomposition, and which (being in rhubarb what peperin is in pepper, or gentianin in gentian,) may be called *rhubarbin.* Besides this, there exists in the extract a brown substance, insoluble in water in its state of purity, but which, when combined with rhubarbin, acquires the property of dissolving, and forms a composition, which is the *caphopicrite* of some chemists, and the *rhubarbine* of Psaff. From this fact, which bears an analogy with that which is found in many other extracts,—for example, in that of the Campeachy wood, where the hematine is united to another principle; or that of the gentian, where the gentianin is combined with a glutinous matter,—M. Caventou concludes that, among vegetable substances, one acts the part of an acid, the other that of an alkali; and that they produce, by their mixture, compounds which assimilate in many respects to saline substances. (*Archives Generales*, November.)

10. *Necessity of Water in the Preparation of Lead-Plaster.*—Attempting to form the lead-plaster, the Emplastrum Plumbi of the Pharmacopœiæ, without the use of water, steam being the source of heat, I was surprised to find, after several hours, during which time the litharge and oil had been kept at a temperature of 220$_{o}$, or thereabout, and constantly stirred, not the slightest appearance of combination. Upon the addition of a small quantity of boiling water, the oil and oxide immediately saponified: water appeared, therefore, to be essential to the formation of the plaster. It also appeared probable the oxide might be in the state of hydrate. To ascertain if such were the case, I precipitated, by potash, the oxide from a quantity of acetate. The precipitate, when washed, was dried by a heat of 220°, until it ceased to lose weight. One hundred grains, heated to redness in a tube, gave off nearly eight grains of water, and assumed the orange-colour of litharge. The recently precipitated oxide was, no doubt, therefore, an hydrate; part of which, with somewhat less than two parts of olive-oil, without any addition of water, at a temperature of 212°, formed in half an hour perfect plaster. Each of these experiments has been repeated with precisely the same results. I am induced to mention this fact, because all pharmaceutical writers limit the action of the water to that of keeping down the temperature.—H. H. (*Journal of Science.*)

---

### MISCELLANEOUS.

11. *Medical, Clerical, and General Life Assurance Society.*—No apology, we apprehend, will be deemed requisite, on our part, for introducing to the notice of such of our readers as may not yet be aware of its existence, some account of an Institution which professes to have for its object the welfare and security of two large classes of the community—the members of the clerical profession, and of our own.

We may, perhaps, be allowed to remark, that our motives in doing so are quite disinterested, as we are not, in any way, personally connected with the undertaking; and further that, in calling to it the attention of our professional brethren, our object is not to invite them to speculation, the *capital* of the Society being already subscribed for, and invested.

The plan of forming a new Life Assurance Company, which should have a more direct reference, than any one previously existing, to the interests of the Medical Profession, was set on foot early in the year 1824, by some eminent practitioners in the metropolis.

These gentlemen, in the progress of their labours, were induced to invite the co-operation of the Clergy; and the zeal and cordiality with which their views were seconded by that learned body, furnished a gratifying proof how highly they appreciated the advantages likely to accrue from the proposed measure.

Under these auspices, the Society was organised, and commenced business in the month of June in the above-mentioned year; and it has, since that period, steadily advanced in character and prosperity.

In confirmation of this statement, we may subjoin an extract from the Report, made to the proprietors, by the Board of Directors, in December last, which is now lying before us:—

"In meeting the friends and early patrons of the Society on the present occasion, the Board of Directors has great satisfaction in being able to state, for their information, that, since the last General Court, the affairs of the Institution have proceeded with a steady and growing prosperity. At that period it was remarked, that the increasing business of the Society warranted the expectation of a progressive improvement; and, perhaps, the most acceptable intelligence that can be now conveyed to the proprietors, will be found in the confirmation of this anticipated result. The business still increases, and the prospect of great ultimate success is more and more favourable. The high respectability of character which the Society has attained, and the peculiar advantages which it possesses, are daily fixing themselves with augmented stability in the public mind.

"It will be satisfactory to the General Court of Proprietors to learn that the shares of the Society appear to be in the hands of permanent holders. Notwithstanding the privilege granted by a former General Court, of reserving five hundred to be placed at the discretion of the Directors, *not a single share* has been so reserved in the office. All of them have been not only distributed, but actually paid for; and they are held by *bona fide* subscribers, as is demonstrated by the fact that no share has hitherto been sold, or even exposed for sale in the market.

"The value of the shares is now increased by the interest that is due upon them; and also by the surplus of the premiums, which is already accumulating, and which the Directors have just cause to hope will, in a reasonable time, form a capital adequate to all the purposes of the Institution, without requiring any additional call upon the proprietors."

It is to the general principle of life assurance, and to the advantageous mode of securing property which is offered to the public by Societies of this kind, that we would wish, upon the present occasion, more particularly to direct the attention of our readers. And it would be difficult, we conceive, to point out, among the improvements which distinguish the civil polity of modern times, any one which has contributed more essentially to the welfare of the community at large, or which has tended to lessen, in a greater degree, the sum of human misery, than the introduction of these establishments.

Yet, widely as their benefits have been diffused, if there be one class of individuals to which, more than another, they may be made availing, it is that composed of *members of the several professions*, whose incomes, the tardy and uncertain reward of anxious, persevering toil, too often cease with the lives of their possessors; while their wives and children, from the refinements of education, and from the sphere of society in which they move, are ill calculated to meet the pressure of adversity.

But, that the practice of life assurance, which affords, of all others, the easiest and most economical method of providing against the contingency to which we have alluded, has not hitherto been duly appreciated,—that, in too many instances, its advantages have been overlooked and neglected,—we have abundant and melancholy proof, not only in the existence of various charities for the relief of the widows and orphans of clergymen and medical men, but likewise in the well-known fact that the premature death, even of men holding a high and distinguished professional rank, has too frequently, from the want of this salutary foresight, plunged their families suddenly, from a state of affluence and all the enjoyments of luxury, into a condition of hopeless poverty and destitution.

Before we take leave of the subject, we may mention, for the information of such of our readers as may not have seen the Prospectus of the "Medical and Clerical Life Assurance Society," that the medical gentlemen officially connected with that Institution are Sir Henry Halford, Sir Astley Cooper, Sir Everard Home, Dr. Bree, Dr. Cholmeley, Dr. Pinckard, Dr. Merriman, Dr. Sutherland, Dr. Ashby Smith, Mr. Earle, Mr. Vance, Mr. Davis, and Mr. Stevenson;—among their clerical coadjutors, we observe the names of several bishops, and other dignitaries of the church.

---

### MONTHLY CATALOGUE OF MEDICAL BOOKS.

### METEOROLOGICAL JOURNAL,

*From January 20, to February 19, 1826, inclusive.*

By Messrs. William Harris and Co. Mathematical Instrument Makers, 50, Holborn, London.

| | MOON. | Rain gauge | THERM. | | | BAROM. | | DE LUC'S HYG. | | WIND. | | ATMOSPHERIC VARIATIONS. | | |
|---|---|---|---|---|---|---|---|---|---|---|---|---|---|---|
| | | | 9 A.M. | MAX. | MIN. | 9 A.M. | 10P.M. | 9 A.M. | 10P.M. | 9 A.M. | 10P.M. | 9 A.M. | 2 P.M. | 10P.M. |
| Jan. | | | | | | | | | | | | | | |
| 20 | | | 37 | 40 | 39 | 30.17 | 30.18 | 89 | 80 | NNW | NNW | Fine | Fine | Fine |
| 21 | | | 40 | 41 | 34 | 30.05 | 30.01 | 82 | 83 | SW | W | | | Foggy |
| 22 | | | 35 | 40 | 31 | 30.09 | 30.06 | 93 | 95 | WSW | W | Foggy | | |
| 23 | ○ | | 36 | 40 | 34 | 30.06 | 30.10 | 95 | 95 | W | NNE | | | |
| 24 | | | 35 | 37 | 34 | 30.28 | 30.30 | 96 | 93 | NE | SW | Fair | | |
| 25 | | | 36 | 37 | 34 | 30.20 | 30.16 | 83 | 93 | SE | E | Fine | | Sl. Fog |
| 26 | | | 36 | 37 | 29 | 30.19 | 30.17 | 83 | 97 | E | ESE | | | Fine |
| 27 | | | 31 | 35 | 32 | 30.14 | 30.14 | 92 | 89 | E | SE | Foggy | | Foggy |
| 28 | | | 36 | 37 | 35 | 30.01 | 30.04 | 85 | 89 | SW | ESE | Fine | | |
| 29 | | | 39 | 45 | 37 | 29.98 | 29.81 | 85 | 82 | SSE | S | | | Fine |
| 30 | ☾ | | 42 | 47 | 42 | 29.67 | 29 60 | 89 | 85 | SSE | SW | | | S. Rain |
| 31 | | .4 | 45 | 47 | 40 | 29.64 | 29.66 | 82 | 86 | SW | SW | | | Fine |
| Feb. | | | | | | | | | | | | | | |
| 1 | | | 46 | 47 | 43 | 29.72 | 29.72 | 86 | 86 | E | SSW | Foggy | | Foggy |
| 2 | | | 47 | 50 | 46 | 29.73 | 29.73 | 85 | 83 | SW | SSW | Fair | | Fine |
| 3 | | .4 | 50 | 52 | 43 | 29.55 | 29.54 | 74 | 84 | SW va. | SW | Fine | | S. Rain |
| 4 | | | 45 | 47 | 42 | 29.76 | 29.75 | 80 | 81 | SW | SSW | | | Cloud. |
| 5 | | | 50 | 50 | 45 | 29.75 | 29.65 | 72 | 78 | SW | SW | | | S. Rain |
| 6 | ● | .12 | 50 | 54 | 43 | 29.45 | 29.65 | 82 | 77 | SW | SW | Rain | Rain | Fine |
| 7 | | | 45 | 48 | 36 | 29.83 | 30.14 | 72 | 70 | W | WSW | Fine | | |
| 8 | | | 39 | 46 | 35 | 30 22 | 30.19 | 82 | 74 | SSW | SSW | | Fine | |
| 9 | | | 38 | 42 | 30 | 30.14 | 30.11 | 84 | 86 | S | SE | | | |
| 10 | | | 31 | 36 | 34 | 30.09 | 30.06 | 89 | 80 | E | SE | Foggy | | |
| 11 | | | 36 | 40 | 38 | 30.01 | 29.93 | 76 | 86 | S | SSE | Fine | | |
| 12 | | | 45 | 47 | 40 | 29.92 | 30.02 | 79 | 87 | SSW | SW | | | |
| 13 | | | 41 | 47 | 41 | 30.02 | 29.84 | 88 | 78 | SW | S | Foggy | | |
| 14 | | .15 | 44 | 45 | 44 | 29.80 | 29.84 | 85 | 88 | SSW | SSE | Rain | Rain | Rain |
| 15 | ☽ | | 45 | 51 | 45 | 29.81 | 29.67 | 88 | 72 | S | S | Fair | Fine | Fine |
| 16 | | | 46 | 50 | 43 | 29.60 | 29.57 | 85 | 80 | SSW | SSW | | | |
| 17 | | | 46 | 47 | 35 | 29.37 | 29.44 | 80 | 78 | SSW | SW | | | |
| 18 | | .20 | 37 | 47 | 37 | 29.64 | 29.75 | 80 | 80 | SW | SSW | Rain | Fair | |
| 19 | | | 45 | 52 | 43 | 29.41 | 29.41 | 85 | 87 | SW | WSW | S. Rain | | Rain |

## Figures and Tables

**Figure f1:**